# Sensitivity of Temperate Desert Steppe Carbon Exchange to Seasonal Droughts and Precipitation Variations in Inner Mongolia, China

**DOI:** 10.1371/journal.pone.0055418

**Published:** 2013-02-05

**Authors:** Fulin Yang, Guangsheng Zhou

**Affiliations:** 1 Key Laboratory of Arid Climatic Change and Reducing Disaster of Gansu Province, Key Open Laboratory of Arid Climatic Change and Disaster Reduction of China Meteorological Administration (CMA), Institute of Arid Meteorology, CMA, Lanzhou, China; 2 State Key Laboratory of Vegetation and Environmental Change, Institute of Botany, Chinese Academy of Sciences, 20 Nanxincun, Xiangshan, Haidian District, Beijing, China; 3 Chinese Academy of Meteorological Sciences, Haidian District, Beijing, China; DOE Pacific Northwest National Laboratory, United States of America

## Abstract

Arid grassland ecosystems have significant interannual variation in carbon exchange; however, it is unclear how environmental factors influence carbon exchange in different hydrological years. In this study, the eddy covariance technique was used to investigate the seasonal and interannual variability of CO_2_ flux over a temperate desert steppe in Inner Mongolia, China from 2008 to 2010. The amounts and times of precipitation varied significantly throughout the study period. The precipitation in 2009 (186.4 mm) was close to the long-term average (183.9±47.6 mm), while the precipitation in 2008 (136.3 mm) and 2010 (141.3 mm) was approximately a quarter below the long-term average. The temperate desert steppe showed carbon neutrality for atmospheric CO_2_ throughout the study period, with a net ecosystem carbon dioxide exchange (NEE) of −7.2, −22.9, and 26.0 g C m^−2^ yr^−1^ in 2008, 2009, and 2010, not significantly different from zero. The ecosystem gained more carbon in 2009 compared to other two relatively dry years, while there was significant difference in carbon uptake between 2008 and 2010, although both years recorded similar annual precipitation. The results suggest that summer precipitation is a key factor determining annual NEE. The apparent quantum yield and saturation value of NEE (NEE_sat_) and the temperature sensitivity coefficient of ecosystem respiration (R_eco_) exhibited significant variations. The values of NEE_sat_ were −2.6, −2.9, and −1.4 µmol CO_2_ m^−2^ s^−1^ in 2008, 2009, and 2010, respectively. Drought suppressed both the gross primary production (GPP) and R_eco_, and the drought sensitivity of GPP was greater than that of R_eco_. The soil water content sensitivity of GPP was high during the dry year of 2008 with limited soil moisture availability. Our results suggest the carbon balance of this temperate desert steppe was not only sensitive to total annual precipitation, but also to its seasonal distribution.

## Introduction

Grassland ecosystems comprise approximately 32% of the global natural vegetation [1], making them important to the global carbon balance. Global climate changes are expected to alter precipitation regimes in grassland biomes [2], where the carbon cycle is particularly sensitive to the amount and timing of precipitation [3]. A recent study shows that grassland ecosystem productivity is sensitive to climate change [4]. Changing precipitation regimes and drought can have a profound impact on carbon fluxes in grassland ecosystems, especially in arid and semi-arid regions characterized by limited water [5], [6]. In temperate grasslands, interannual variability in total precipitation is the primary climatic factor that causes fluctuations in net annual primary production [7]–[9] and net ecosystem carbon dioxide exchange (NEE) [10]. Studies on various grassland ecosystems extensively supported the positive relationship between annual NEE and total annual precipitation [11]–[16]. Depending on the amount of annual precipitation, a grassland ecosystem can switch from being a carbon sink in the wet or normal years to a net carbon source in the drought years [12], [13]. However, other studies have shown that the interannual precipitation distribution can alter carbon uptake and release regardless of the total annual precipitation [8], [16], [17].

Drought is a common factor that limits vegetation growth and ecosystem carbon uptake in semi-arid grasslands. Droughts are related to lower annual rainfall and to different rainfall distributions [18]. The asymmetric distribution of seasonal precipitation can lead to intermittent droughts. Drought spells can substantially modify the seasonal development of leaf area and change plant physiology [19], making them have a large impact on the ecosystem as sources or sinks of atmospheric CO_2_ [20]. An extreme drought in Europe in 2003 caused many ecosystems to lose carbon [21]. Aires *et al.* [11] found that winter and early spring droughts suppressed grass production and canopy development, consequently decreasing the maximum daily NEE of the Mediterranean C3/C4 grassland in southern Portugal significantly during the dry year. Hussain *et al.* [22] showed that the leaf area index (LAI) reduction caused by a summer drought decreased the gross primary production (GPP) and the ecosystem respiration (R_eco_) in a temperate grassland in Germany. Flanagan *et al.* [12] reported that fall droughts may accelerate leaf fall and shorten the growing season, consequently decreasing the seasonal cumulative GPP. Scott *et al.* [23] suggested that severe droughts can lead to a change in plant community structure, and that NEE was suppressed during the drought years in a semi-desert grassland in the USA. Drought conditions affect the terrestrial carbon balance by modifying the rates of and the coupling between the carbon uptake by photosynthesis (GPP) and release by R_eco_ [24], [25]. Reichstein *et al.* [18] suggested that drought conditions may have different effects on plant assimilation and ecosystem respiration, and that short-term drought will suppress R_eco_ before affecting GPP.

Photosynthesis is an important factor in regulating R_eco_ in diurnal [26], daily [11], [17], seasonal, and yearly timescales [27]. Davidson *et al.* [28] suggested that gross photosynthesis can control the substrate availability of autotrophic and heterotrophic respiration through root exudates. However, Yan *et al.* [29] reported that water can regulate the effects of the photosynthetic substrate supply on soil respiration in a semi-arid steppe. Insufficient information is available about whether a strong correlation between GPP and R_eco_ holds true in a desert steppe ecosystem, where soil water availability is considered as the factor that limits vegetation growth the most.

Desert steppes with annual precipitation between 150 mm and 250 mm are the most arid grassland ecosystems [30] located in the transitional zones between steppes and deserts [31]. The 17.5 million ha temperate desert steppe area in China provided 0.066 Pg of carbon storage in its biomass [32]. Recent studies have shown that the annual mean temperature and interannual precipitation variability increased and the spring and winter precipitation decreased over the past 40 years [33]. The low soil moisture availability in desert steppes associated with temperature increase may intensify water limitation effects on carbon flux.

In this study, eddy covariance technology was used to continuously measure NEE over the Inner Mongolian temperate desert steppe from 2008 to 2010. The objectives of this study were as follows: (1) to examine the seasonal and interannual variability in GPP, R_eco_, and NEE; (2) to elucidate environmental and physiological regulations on carbon flux components; and (3) to evaluate the seasonal distribution and the total amount of precipitation that affect the carbon balance over a temperate desert steppe in Inner Mongolia, China.

## Materials and Methods

### 1.1. Ethics Statement

All observational and field studies at the desert steppe were undertaken with relevant permissions from the owners of private land: Mr. L.S. Chai. The field studies did not involve endangered or protected species, and the location was not protected in any way during the study period.

### 1.2. Study Site

The study site is located north of the Sunitezuoqi County, Inner Mongolia Autonomous Region, China (44°05′N, 113°34′E, 970 m a.s.l.), and it is classified as temperate desert steppe. The plant community is dominated by the bunch grass *Stipa klemenzii* and the herb *Allium polyrrhizum*. The grass canopy is 0.20 m to 0.35 m tall during mid-summer. The study site was fenced in August 2007 to prevent grazing and other disturbances. The soil in the area was classified as brown calcic with an average bulk density of 1,630 kg m^−3^. The study site has a mean air temperature of 3.2°C and mean annual precipitation of 183.9±47.6 mm (from 1965 to 2004, Sunitezuoqi Weather Station). Most of the precipitation (85%) falls between May to September. The area has an arid to semi-arid temperate continental climate.

### 1.3. Eddy Covariance Measurements

An open-path eddy covariance (EC) system was installed at the study site to measure the net ecosystem exchange of CO_2_ and latent heat flux at a measurement height of 2.0 m. The EC system included a 3D sonic anemometer–thermometer (CSAT-3, Campbell Scientific Inc., Logan, UT, USA) and an open path infrared gas (CO_2_/H_2_O) analyzer (LI-7500, LI-COR Inc., Lincoln, NE, USA). Raw signals were recorded at 10 Hz using a data logger (CR5000, Campbell Scientific Inc., Logan, UT, USA).

### 1.4. Meteorology, Soil and Biotic Factor Measurements

A meteorological tower was situated near the EC tower to measure environmental variables. Air temperature (Ta) and relative humidity (RH) were measured at a height of 2.0 m (HMP45C, Vaisala, Helsinki, Finland). A horizontal wind speed sensor (014A, Campbell Scientific Inc., Logan, UT, USA) was situated at a height of 2.0 m to measure horizontal wind speed (Ws). Photosynthetically active radiation (PAR) and net radiation (Rn) were measured at 2.4 m above the ground by using a quantum sensor (LI-190SB, LI-COR Inc., Lincoln, NE, USA) and a four-component net radiometer (CNR-1, Kipp & Zonen, Delft, Netherlands), respectively. Precipitation was measured above the canopy by using a tipping bucket rain gauge (Model 52203, RM Young Inc., Traverse City, MI, USA). Soil temperature (Ts) at 0.05 m underground was measured using a thermistor (107L, Campbell Scientific Inc., Logan, UT, USA). The soil water content (SWC) at a depth of 0.10 m was measured using time-domain reflectometry probes (CS616, Campbell Scientific Inc., Logan, UT, USA). Two soil heat plates (HFP01, Hukeflux Inc., Delft, Netherlands) were used to measure the soil heat flux (G) at 0.08 m below the soil surface in separate locations. All meteorological and soil sensors were sampled every 2 s, and were stored as a half-hour means by a data logger (CR23X, Campbell Scientific Inc., Logan, UT, USA).

Biomass sampling was conducted monthly in the growing season by clipping eight 1 m ×1 m quadrats. In each quadrat, all the plants were cut at the ground surface and then oven dried at 65°C to a constant weight. The dominant species leaf area ratio (m^2^ g^−1^) was also measured. The total LAI (m^2^ m^−2^) was estimated by means of specific leaf area (m^2^ g^−1^) multiplied by biomass (g m^−2^) [31]. The monthly samplings of biomass and LAI determination were performed from 2008 to 2009.

### 1.5. Data Processing and Flux Computation

Raw data from the eddy-covariance measurements were obtained using the EdiRe software (www.geos.ed.ac.uk/abs/research/micromet/EdiRe: developed by University of Edinburgh, UK). The CO_2_ fluxes were determined by the eddy covariance method as the mean covariance between fluctuations in vertical wind speed (*w’*) and the carbon dioxide concentration (*c’*) on a half-hourly basis (Eq. 1) [10].
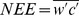
(1)


Negative NEE denotes the net carbon uptake of the ecosystem. Prior to the scalar flux computation, spikes exclusion, two-dimension coordinate rotation and air density fluctuations correction [34] were performed [31]. The CO_2_ storage term was not included in NEE computation because CO_2_ concentration profile was not measured.

Half-hourly flux data were rejected following these criteria: (1) incomplete half-hourly measurement mainly caused by power failure, IRGA calibration; (2) rain events; (3) outliers [35]; and (4) low-turbulence conditions. The moving point test (MPT) was used to determine the friction velocity threshold (u*_c_) for nighttime CO_2_ flux under stable atmospheric conditions [36], [37], suggesting the u*_c_ was 0.11 m s^−1^ for this desert steppe ecosystem. The nighttime NEE data with lower than u*_c_ was discarded. Negative nighttime CO_2_ fluxes occurred prevalently during the non-growing season in this study, which may be due to the instrument LI-7500 surface heating effect. We tried to conduct Burbra correction for the non-growing season data [38]. However, the corrected data showed large noise and unrealistic flux values. The Burba correction did not work in this study. We discarded the negative CO_2_ fluxes during 1 January to 30 April and 16 October to 31 December, likely to offset the non performance of the self-heating correction, which anyway did not affect the reliability of the EC flux measurement [39], [40].

Due to missing and discarded data, the data gaps during the whole growing period were 36.5% of total data. To derive a continuous time series of NEE, the data gaps of less than 2 h were filled using linear interpolation, and the other data gaps were filled using the look-up table method. If meteorological data was absent, mean daily variation gap-fillings were used [41], [42].

The energy balance ratio (EBR) was used to assess the accuracy of the eddy covariance measurements. For a short-statured canopy, EBR can be calculated using the following equation based on the half-hourly dataset after quality controls:
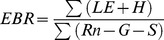
(2)where LE and H are the latent and sensible heat fluxes (W m^−2^); Rn is net radiation (W m^−2^); G is the soil heat fluxes (W m^−2^); and S is the soil heat storage (W m^−2^) which can be estimated by Ts and SWC [43]. EBR was 0.9 for the entire observation period, indicating that (H+LE) was close to (Rn-G-S). The flux measurement performance of eddy covariance system was acceptable. So we assumed that the advective losses of energy and CO_2_ were small, and energy balance closure correction to CO_2_ flux was neglected.

As we had only one eddy covariance tower, the daily-differencing approach [44] was used to estimate the uncertainty in annually integrated NEE. The identical environmental conditions (mean half-hourly PAR within 50 µmol photons m^−2^ s^−1^, Ta within 2°C, and SWC within 0.5%) were adopted to calculate the standard deviation of the difference.

The partitioning of NEE into GPP and R_eco_ was based on stepwise procedures and algorithms of Reichstein *et al.* [45]. The equation of Lloyd and Taylor [46] was used to describe the response of half-hourly nighttime R_eco_ to soil temperature (Eq. 3):
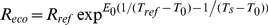
(3)where E_0_ is the activation energy (K); T_ref_ is set to 10°C; R_ref_, the reference ecosystem respiration at T_ref_; T_0_ is a constant (−46.02°C) and Ts is the soil temperature. Consistent temperature sensitivity between daytime and nighttime was assumed for daytime R_eco_ calculations. The daily R_eco_ was taken as the sum of daytime and nighttime R_eco_. GPP was calculated as follows:
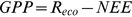
(4)


### 1.6. Canopy Surface Conductance

Based on the inverted Penman-Montieth equation, the half-hourly canopy surface conductance (*g_c_*, m s^−1^) was calculated as follows [47]:
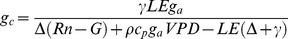
(5)where *γ* is the psychrometric constant (kPa °C^−1^), LE is the latent heat flux (W m^−2^), Δ is the slope of the saturation water vapor pressure over the air temperature curve (kPa °C^−1^), Rn is the net radiation (W m^−2^), G is the soil heat flux (W m^−2^), *ρ* is the air density (kg m^−3^), *C_p_* is the specific heat of air (J kg^−1^°C^−1^), and VPD is the air vapor pressure deficit (kPa), and *g_a_* is the aerodynamic conductance of the air layer between the canopy and the flux measurement height (m s^−1^). Using the Monteith-Unsworth equation, *g_a_* was obtained as follows [48]:
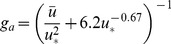
(6)where 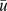 is the mean wind speed (m s^−1^) at a height of 2 m and u* is the friction velocity (m s^−1^). gc was excluded based on the following criteria: (1) rain events, (2) nighttime data (with incident solar radiation values less than 20 W m^−2^), (3) low turbulence (u*<0.2 m s^−1^), and (4) anomalies.

### 1.7. Data Analysis

#### 1.7.1. Carbon flux response to PAR and Ts

The relationship between the daytime (with incident solar radiation values greater than 20 W m^−2^) NEE (µmol CO_2_ m^−2^ s^−1^) and PAR (µmol photons m^−2^ s^−1^) was assessed using a Michaelis-Menten rectangular hyperbola equation [41]:
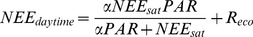
(7)where *α* is the apparent quantum yield or the initial slope of the light response curve (µmol CO_2_ µmol^−1^ photons), NEE_sat_ is the value of the NEE at a saturating light level, and R_eco_ is a bulk estimate of the ecosystem respiration.

The relationship between the nighttime NEE or R_eco_ (µmol CO_2_ m^−2^ s^−1^) and soil temperature at a 5 cm depth (Ts, °C) was calculated using the Van’t Hoff equation [11], [31]:
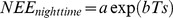
(8)where *a* and *b* are the regression parameters. The temperature sensitivity coefficient (Q_10_) of R_eco_ was determined as follows:
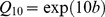
(9)


#### 1.7.2. Statistical analysis

Regression analysis and model parameter fitting were performed using the statistical package SigmaPlot 10.0 software (Systat Software Inc. San Jose, CA, USA). Other data computation and analysis were conducted using MATLAB 7.9 software (MathWorks Inc., Natick, MA, USA).

## Results

### 2.1. Environmental Variables

**Figure 1:**
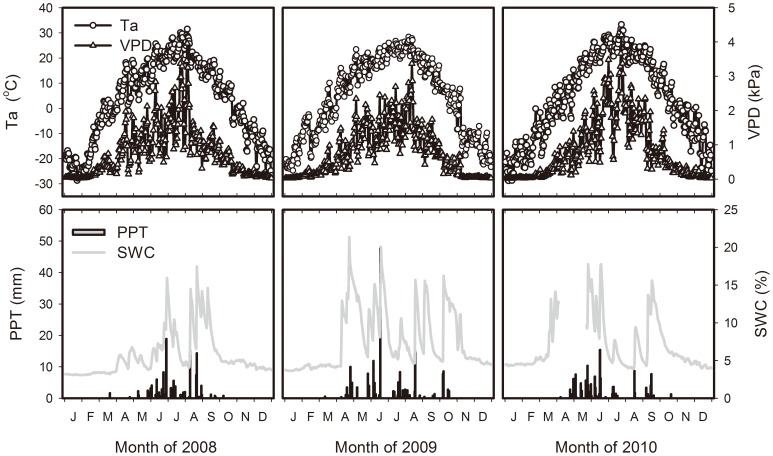
Seasonal variation in daily average temperature (Ta) and vapor pressure deficit (VPD), and daily precipitation (PPT) and daily average soil water content (SWC) throughout the study period. Soil water content data from 6 April to 23 May were omitted because of a malfunction in the connecting cable between the soil moisture sensor and the data logger.

The variations in weather conditions from 2008 to 2010 (Figure 1) recorded by the Sunitezuoqi weather station (1965 to 2004) are summarized in Table 1. The annual precipitation in 2009 (186.4 mm) was close to the average precipitation (183.9 mm) recorded by the Sunitezuoqi weather station, whereas the annual precipitations in 2008 (136.3 mm) and 2010 (141.3 mm) were approximately a quarter below the average. The amount of precipitation received during the growing seasons (May to September) was close among the three study years. However, the timing of the rainfall events differed significantly among the three years. Over 65% of the total annual precipitation fell in May and June 2010, while only 44.6% and 47.5% fell during the same periods in 2008 and 2009, respectively. The amount of precipitation in July and August of 2008, 2009, and 2010 were 73.9%, 52.3%, and 23.9% of the long-term average in the same period, respectively. The temperate desert steppe experienced much drought during the critical period, July and August, for plant development and growth in the measured years, especially in 2010, which had the longest severely dry period. Less than 5 mm of precipitation was experienced in September 2008 and 2009. On the other hand, September 2010 experienced six times more precipitation than September 2008 and 2009.

**Table 1:** Comparison of environmental conditions in temperate desert steppe, Inner Mongolia, during 2008 to 2010.

	Year	May	June	July	August	September	May to September	Annual
PPT	2008	12.6	48.3	23.6	45.0	2.7	132.2	136.3
	2009	18.4	70.2	23.5	25.1	3.2	140.4	186.4
	2010	51.2	40.7	11.2	11.0	20.4	134.5	141.3
	Mean	27.4	53.1	19.4	27.0	8.8	135.7	154.7
	1965 to 2004	16.2	28.4	48.3	44.5	18.6	155.9	183.9
Ta	2008	11.6	19.4	23.9	20.5	13.6	17.8	3.1
	2009	15.1	17.9	22.3	21.4	13.3	18.0	2.4
	2010	13.5	22.1	25.6	20.6	14.8	19.3	2.0
	Mean	13.4	19.8	23.9	20.8	13.9	18.4	2.5
	1965 to 2004	13.7	19.4	22.1	20.1	13.3	17.7	3.2
SWC	2008	4.8	7.6	7.7	9.7	8.9	7.7	5.6
	2009	10.8	12.4	8.6	7.8	8.8	9.7	7.7
	2010	13.7	11.3	5.5	5.6	10.2	8.4	6.8
	Mean	9.8	10.4	7.3	7.7	9.3	8.6	6.7

PPT, precipitation (mm); Ta, air temperature (°C); SWC, soil water content (%).

Daily Ta varied dramatically across the study period, and ranged from −33.4°C to 32.9°C. The annual temperature in 2008 was close to the long-term average temperature, whereas the annual temperatures in 2009 and 2010 were below the long-term average temperature, due to the relatively low temperature during non-growing seasons. However, in June and July 2010, a distinct hot spell occurred during the growing season, resulting in the highest VPD in June and July as compared to the VPD during the same period in 2008 and 2009.

The SWC was related to the precipitation pattern during the growing seasons and ranged from 3.8% (27 May 2008) to 20.1% (19 June 2009). Peak daily SWC values were obtained approximately a day after rain events. The SWC in 2009 was the highest among the three study years. In 2008, the temperate desert steppe experienced two significant dry periods. Only 9.6 mm of precipitation was recorded from 17 May to 1 June (16 days), with an average SWC of 4.1%. The next dry period occurred from 25 July to 8 August (15 days), with a low average SWC of 4.5%. In 2010, the seasonal severe drought started from 24 July to 16 August (24 days), when only 0.2 mm of precipitation was recorded, with a low averaged SWC of 4.2%. The dry period occurred exactly at the normal vegetation peak growth stage. Soil drying continued for approximately a month, with a minimum SWC of 3.9% on 14 August 2010.

**Figure 2:**
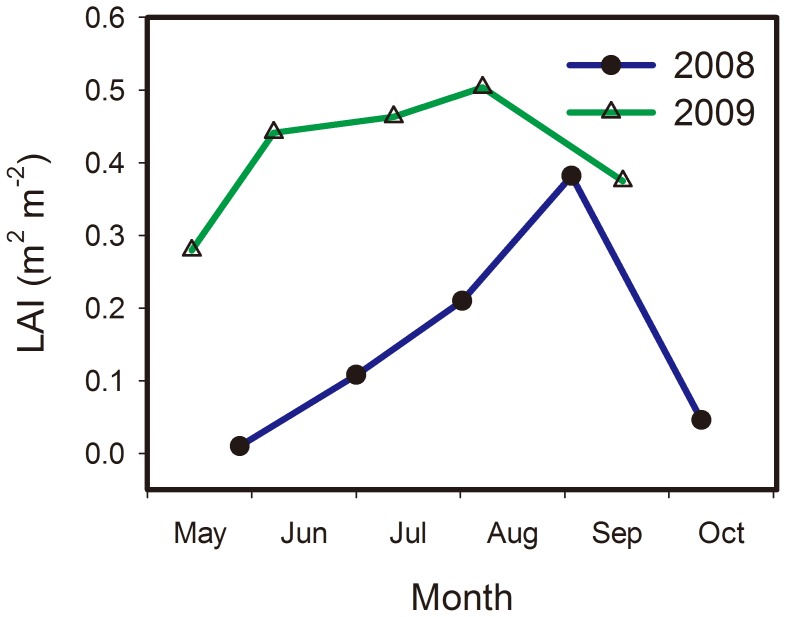
Seasonal dynamics of leaf area index (LAI) in 2008 and 2009.

The maximum LAIs were 0.38 and 0.50 m^2^ m^−2^ in 2008 and 2009, respectively, and occurred during the peak growth period. The LAI in every growth period of 2009 was generally higher than that in 2008 (Figure 2).

### 2.2. Seasonal Variations in the Cumulative GPP, R_eco_, and NEE

**Figure 3:**
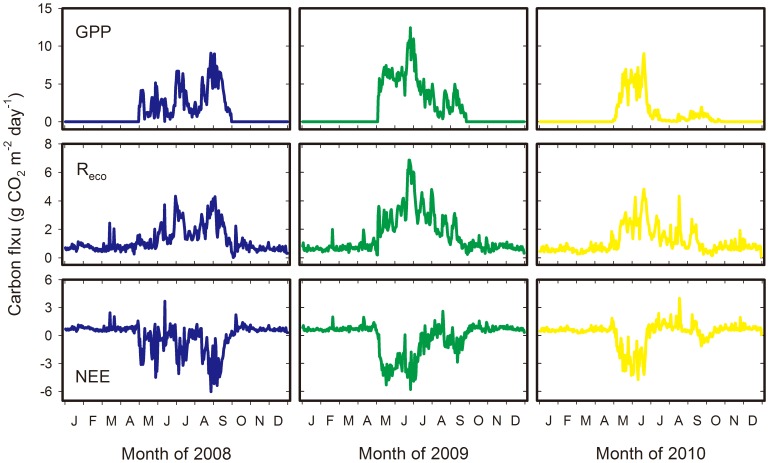
Seasonal variations in daily integrated net ecosystem carbon exchange (NEE), gross primary production (GPP), and ecosystem respiration (R_eco_) over the course of the study. Negative NEE denotes the net carbon uptake of the ecosystem.

Strong seasonal variations in GPP, R_eco_, and NEE of the temperate desert steppe ecosystem were observed. The seasonal variations in 2008 were larger compared with those in 2009 and 2010 (Figure 3). The temperate desert steppe continuously lost carbon via soil respiration at low rates during the non-growing seasons of the three study years. The net carbon uptake began in early May (1 May 2008; 5 May 2009 and 2010), when the daily air temperature at a height of 2 m exceeded ca. 10°C. The seasonal variation in carbon fluxes during the growing seasons varied throughout the three study years. Only 19% of the days in 2010 (70 days) had a net sequestration of carbon, while 31% of the days in 2008 (115 days) and 2009 (113 days) had carbon gain. The maximum daily rates of GPP and R_eco_ in 2009 were 12.4 and 6.9 g CO_2_ m^−2^ day^−1^, respectively, and were higher compared with those in the dry years of 2008 and 2010. The maximum daily NEEs in 2008 and 2009 were both approximately −6.0 g CO_2_ m^−2^ day^−1^, whereas that in 2010 was only −4.8 g CO_2_ m^−2^ day^−1^.

In terms of the season dynamics, significant differences were evident in the CO_2_ exchange of the three study years (Figure 3 and Table 2). In 2008, the CO_2_ fluxes over the temperate desert steppe tracked closely with rain events and soil moisture. The ecosystem switched from being a net carbon sink to a source and then went back to a sink for time periods linked to precipitation processes in the growing season. The ecosystem became a daily carbon sink (with a negative NEE) in early May. However, both GPP and R_eco_ significantly decreased during the three drought spells that occurred in the growing season. GPP was lower than the R_eco_ and resulted in positive NEEs during the drought periods. The maximum net CO_2_ uptake period occurred in the early growth season of 2009, and not in 2008, because of the higher amounts of rainfall (88.6 mm during May and June 2009) that was experienced during the period. The ecosystem experienced a severe drought from late July to mid-August 2009. GPP decreased at an even higher rate and turned the ecosystem into a weak carbon source (8.3 g CO_2_ m^−2^ carbon release during 29 June to 18 August). Although the ecosystem shifted to carbon sink again across the severe drought period, notable carbon uptake characteristics that are similar to those evident in 2008 were not exhibited. Compared to 2008 and 2009, the periods of negative NEE in 2010 were smaller in magnitude and spanned shorter durations because of the severe summer drought, in which the carbon uptake was focused in May and June. The ecosystem suffered considerable seasonal droughts from July to August. The severe water stress (daily SWC lower than 4.5%) resulted in a GPP that was close to zero. The temperate desert steppe ecosystem became a carbon source during the dry period, except for some parts of September when carbon uptake occurred because of the spark rainfall.

**Table 2:** Comparison of carbon fluxes in temperate desert steppe, Inner Mongolia, during 2008 to 2010.

	Year	May	June	July	August	September	Annual
GPP	2008	19.1	14.0	29.3	33.8	33.9	130.0
	2009	42.9	58.5	42.5	18.1	16.2	178.3
	2010	29.9	40.9	5.0	2.4	7.1	87.7
	Mean	30.6	37.8	25.6	18.1	19.1	132.0
R_eco_	2008	8.2	14.9	18.9	20.9	17.5	122.7
	2009	18.7	33.0	30.1	19.7	11.2	155.4
	2010	16.0	24.5	14.1	11.3	9.9	113.8
	Mean	14.3	24.1	21.0	17.3	12.9	130.6
NEE	2008	−10.8	0.9	−10.3	−12.9	−16.3	−7.2
	2009	−24.2	−25.5	−12.5	1.5	−5.1	−22.9
	2010	−13.9	−16.4	9.1	8.9	2.8	26.0
	Mean	−16.3	−13.7	−4.6	−0.8	−6.2	−1.4
R_eco_/GPP	2008	0.43	1.06	0.65	0.62	0.52	0.94
	2009	0.44	0.56	0.71	1.09	0.69	0.87
	2010	0.54	0.60	2.82	4.71	1.39	1.30
	Mean	0.47	0.74	1.39	2.14	0.87	0.99

GPP, the gross primary production (g C m^−2^); R_eco_, the ecosystem respiration (g C m^−2^); NEE, the net ecosystem carbon exchange (g C m^−2^); R_eco_/GPP, the ratio of R_eco_ to GPP.

**Figure 4:**
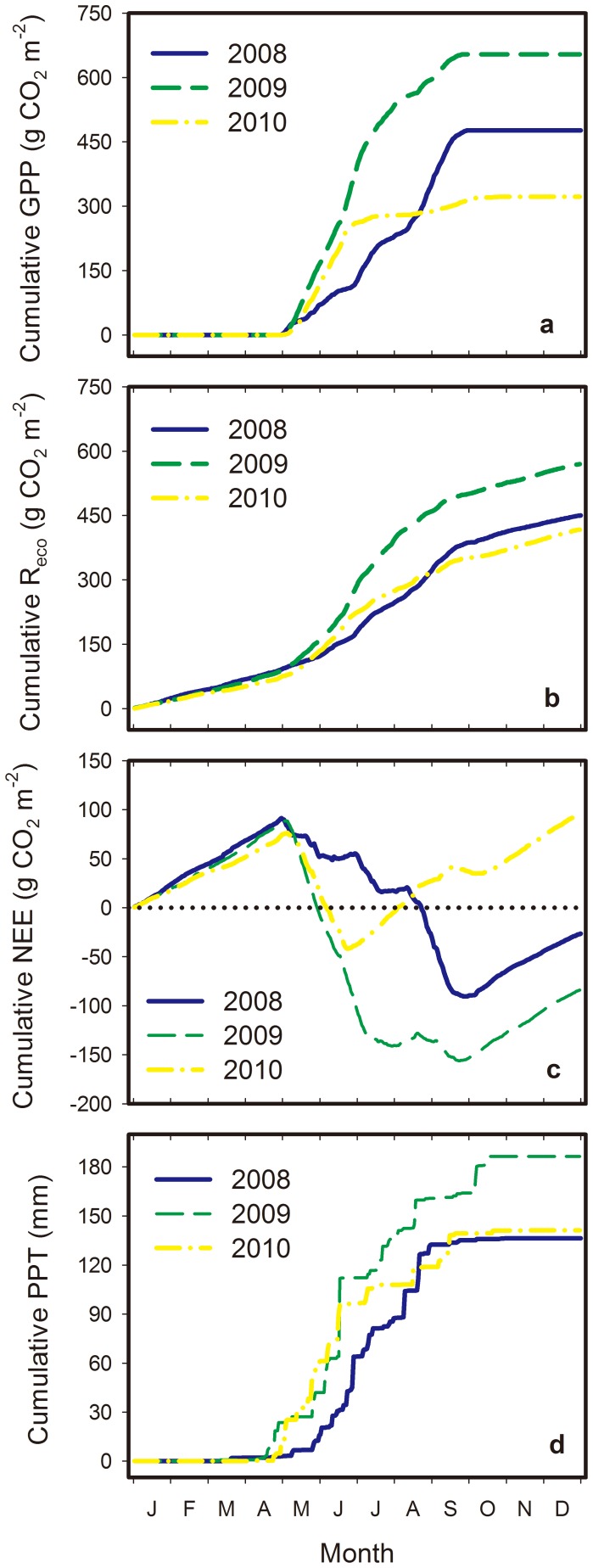
Yearly cumulative net ecosystem carbon exchange (NEE), gross primary production (GPP), ecosystem respiration (R_eco_), and precipitation (PPT) in the Inner Mongolia temperate desert steppe from 2008 to 2010. Negative NEE indicates that the ecosystem is gaining carbon.

Figure 4 shows the seasonal evolutions of the cumulative GPP, R_eco_, and NEE over the three study years. The desert steppe ecosystem exhibited a neutrality (−4.1 g C m^−2^) throughout the study period (from January 2008 to December 2010). The uncertainty in annual NEE measurements inferred from daily-differencing approach was ±12.4, ±11.9, and ±10.0 g C m^−2^ yr^−1^ in 2008, 2009, and 2010, respectively. Compared with the two relatively dry years of 2008 and 2010, the ecosystem fixed more carbon during 2009 (Figure 4a), but with greater R_eco_ (Figure 4b). Based on the cumulative annual NEE data, the temperate desert steppe was a weak carbon sink during 2008 and 2009, while it was a weak carbon source during 2010 (Figure 4c). Although similar amounts of rainfall were recorded during the dry years of 2008 and 2010, the ecosystem fixed more carbon with 33.2 g C m^−2^ in 2008 than in 2010. The differences in carbon budget were primarily caused by the smaller GPP value from July to September 2010. Moreover, the respiration in 2010 was less than that in 2008 (Table 2).

### 2.3. Response of Carbon Flux to Environmental Variables

#### 2.3.1. Response of NEE to PAR

The relationship between the daytime NEE and PAR from May to September of the three study years was described using the Michealis-Menten model (Eq. 7) based on half-hourly data. The rectangular hyperbolic (Eq. 7) produced a good fit to the data when PAR was less than 1,600 µmol photons m^−2^ s^−1^ (approximately the light saturation point). The values of *α* and NEE_sat_ in 2009 were larger compared with those in 2008 and 2010, with the lowest value observed in 2010. The *α* value in 2009 was thrice of that in 2010, and the NEE_sat_ value in 2009 was twice of that in 2010. The NEE_sat_ and *α* values in 2008 were also larger compared with those in 2010, although the averaged SWC in 2010 was slightly higher than that in 2008. The NEE_sat_ and *α* values for the three integrated study years were −2.3 (±0.01) µmol CO_2_ m^−2^ s^−1^ and −0.006 (±0.001) µmol CO_2_ µmol^−1^ photons, respectively (Table 3).

**Table 3:** Mean soil water content and the parameters used to describe the rectangular hyperbolic response of daytime net ecosystem CO_2_ exchange to photosynthetically active radiation during May to September of the three measured years as described in Eq. 7.

Years	SWC	*α*	NEE_sat_	R_eco_	*n*	*R^2^*	*P-value*
2008	7.8	−0.006 (±0.001)	−2.6 (±0.1)	0.5 (±0.1)	32	0.94	<0.0001
2009	9.5	−0.010 (±0.003)	−2.9 (±0.2)	0.8 (±0.2)	32	0.94	<0.0001
2010	8.5	−0.003 (±0.002)	−1.4 (±0.1)	0.3 (±0.2)	32	0.75	<0.0001
Three years	8.6	−0.006 (±0.001)	−2.3 (±0.1)	0.5 (±0.1)	32	0.95	<0.0001

SWC, soil water content (%); *α*, the apparent quantum yield (µmol CO_2_ µmol^−1^ photons); NEE_sat_, the saturation value of NEE at an infinite light level (µmol CO_2_ m^−2^ s^−1^); R_eco_, the model-derived bulk ecosystem respiration (µmol CO_2_ m^−2^ s^−1^); *n*, the number of samples.

#### 2.3.2. Response of GPP to *g_c_*

**Figure 5:**
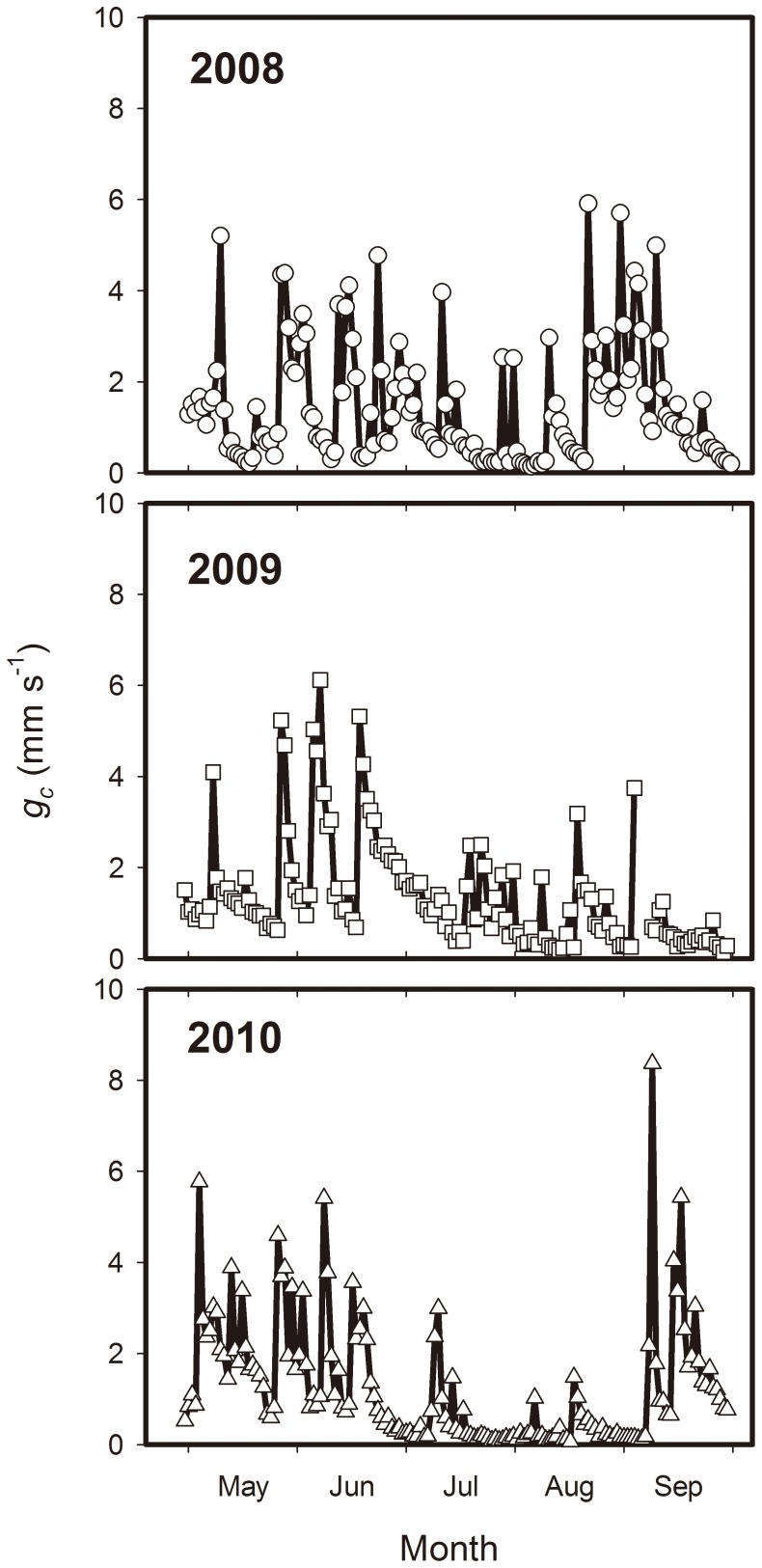
Temporal variations in canopy surface conductance (*g_c_*) from May to September.

**Figure 6:**
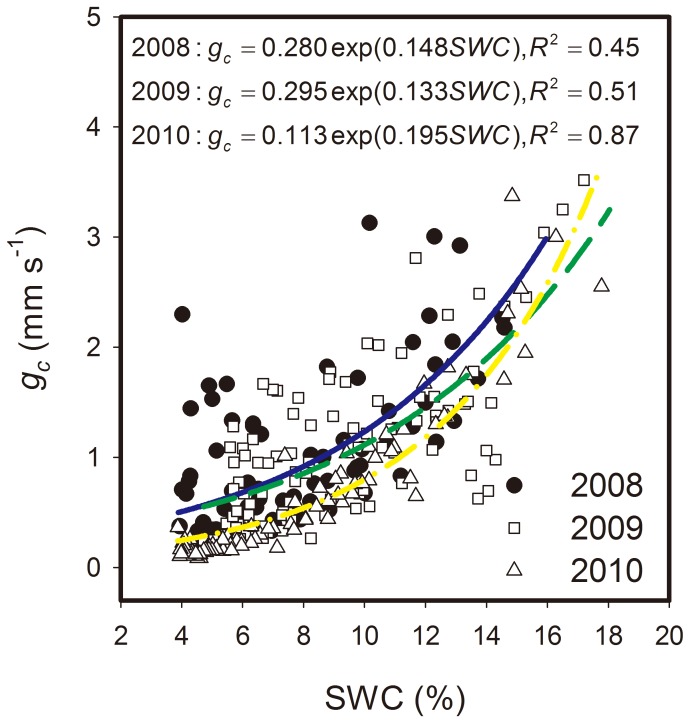
Exponential relation between canopy surface conductance (*g_c_* and soil water content (SWC). Data were obtained from May to September of each of the three study years. Rainy days were excluded from the analysis.

The daily *g_c_* values at the study site were consistently low and ranged between 0.06 mm s^−1^ (17 August 2010) and 8.37 mm s^−1^ (9 September 2010) from May to September in the three study years (Figure 5). The season variance of *g_c_* was relatively small in 2008, except for the low *g_c_* value in July. *g_c_* during the early growth season (May and June) was higher than that in the later growth season (September) of 2009, whereas the *g_c_* during the early growth season (May and June) and later growth season (September) was higher than that in the mid growth season (July and August) of 2010. The seasonal variance of *g_c_* was related to the vegetation development and the differences in soil moisture or precipitation. The *g_c_* value fluctuated closely with the drying or wetting of the surface soil and decreased with the depletion of soil moisture. Daily mean *g_c_* values were significantly correlated with the SWC in an exponential manner (*P*<0.01) from May to September of the three study years (Figure 6).

**Figure 7:**
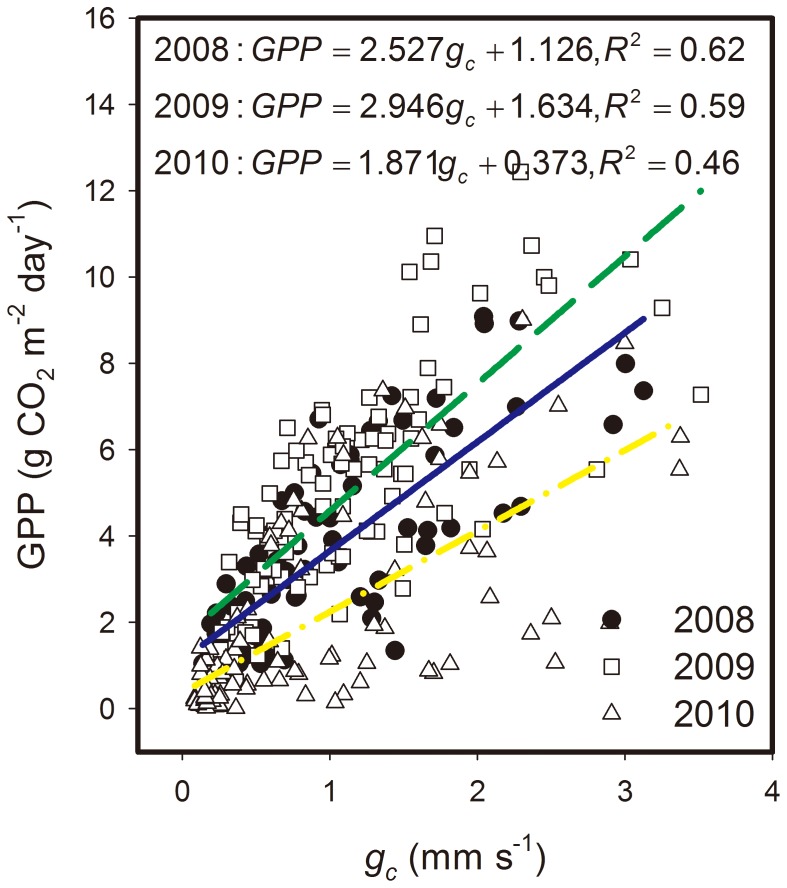
Linear relation between daily gross primary production (GPP) and canopy surface conductance (*g_c_*). Data were obtained from May to September of each of the three study years. Rainy days were excluded from the analysis.

*g_c_* strongly controlled GPP over the temperate desert steppe, and GPP increased linearly with increasing *g_c_* (*P*<0.01), as shown in Figure 7. Slopes of GPP-*g_c_* relationships during the growing seasons significantly varied among the three study years. The slope (2.9±0.3) of GPP-*g_c_* regression line in the 2009 growing season was the highest, while GPP-*g_c_* regression line in 2010 had the lowest slope value (1.9±0.2) among the three study years. These results suggest that the *g_c_* sensitivity of GPP in 2009 was higher than that in 2010, whereas the *g_c_* sensitivity of GPP in 2008 was in the middle range.

#### 2.3.3. Response of GPP and R_eco_ to soil moisture

**Figure 8:**
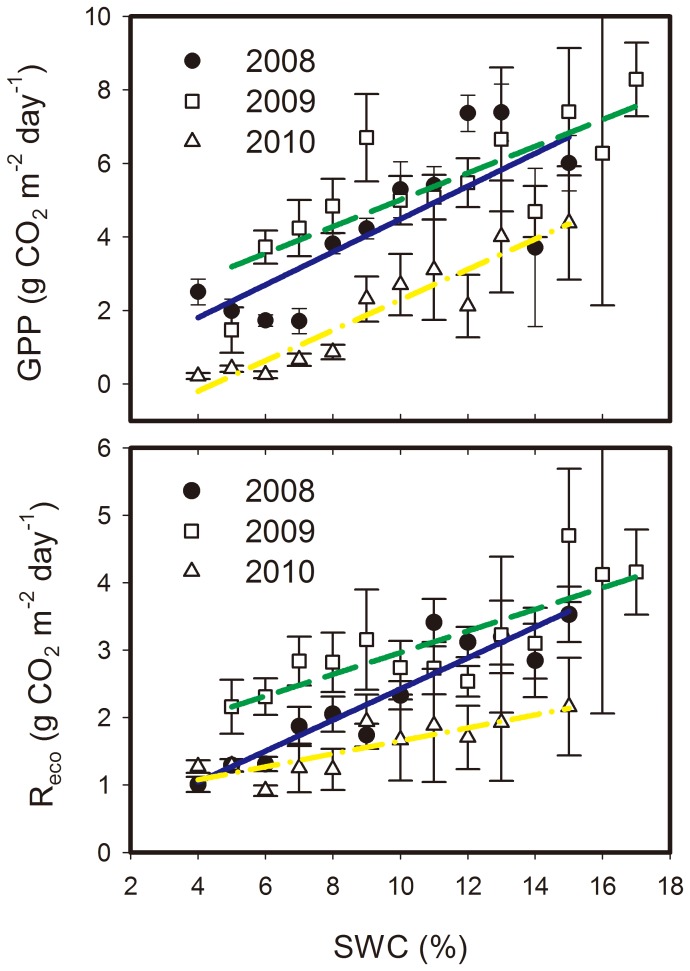
Responses of gross primary productivity (GPP) and ecosystem respiration (R_eco_) to soil water content (SWC). GPP and R_eco_ data from May to September were averaged with a bin width of 1% for SWC. Error bars represent one standard error. Rainy days were excluded from the analysis.

Figure 8 illustrates the responses of daily GPP and R_eco_ to SWC during May to September of the three study years. Daily GPP and R_eco_ values decreased linearly with increasing soil moisture stress. For each observation year, the SWC sensitivity of R_eco_ was less than the SWC sensitivity of GPP, whereas the slope of R_eco_-SWC regression lines was much lower than that of GPP-SWC responding lines (Table 4). Throughout the study period, the SWC sensitivity of GPP in 2009 was less than that in 2008 and 2010, while the SWC sensitivity of R_eco_ in 2008 was greater than that in 2009 and 2010 (Table 4).

**Table 4:** The regression equations between gross primary productivity and soil water content, and between ecosystem respiration and soil water content in the three years.

Year	Equations	*n*	*R^2^*	*P-value*
2008	GPP = 0.45 (±0.11) SWC +0.02 (±1.13)	12	0.61	*P* = 0.003
	R_eco_ = 0.23 (±0.03) SWC +0.12 (±0.29)	12	0.87	*P*<0.001
2009	GPP = 0.36 (±0.08) SWC +1.37 (±0.94)	13	0.65	*P*<0.001
	R_eco_ = 0.16 (±0.03) SWC +1.35 (±0.39)	13	0.68	*P*<0.001
2010	GPP = 0.41 (±0.05) SWC − 1.85 (±0.47)	11	0.89	*P*<0.001
	R_eco_ = 0.10 (±0.02) SWC +0.69 (±0.19)	11	0.72	*P*<0.001

GPP, gross primary productivity (g CO_2_ m^−2^ day^−1^); SWC, soil water content (%); R_eco_, ecosystem respiration (g CO_2_ m^−2^ day^−1^).

#### 2.3.4. Response to R_eco_ to soil temperature and GPP

The response of half-hourly nighttime NEE (R_eco_) to soil temperatures less than 25°C from May to September of the three study years was analyzed, based on the Van’t Hoff equation. The NEE data were averaged with Ts bins of 1°C. The regression coefficients of the fitted curves for the different years are presented in Table 5. For the integrated three growing seasons of the measured years, the temperature sensitivity coefficient (Q_10_) was 2.1, with variance in the different years. In this study, high Q_10_ value (2.3) was observed in 2009 when the desert steppe received the most rainfall, whereas low Q_10_ value (1.8) occurred in 2010 with the long drought season (Table 5).

**Table 5:** Regression coefficients as described in Eqs. 8–9, lower than 25°C.

	SWC	a	b	R^2^	Q_10_	P-value
2008	7.7	0.005	0.07	0.81	2.1 (±0.2)	<0.001
2009	10.1	0.005	0.08	0.91	2.3 (±0.2)	<0.001
2010	9.8	0.007	0.06	0.58	1.8 (±0.2)	<0.001
Three years	9.0	0.006	0.07	0.84	2.1 (±0.2)	<0.001

SWC, soil water content (%); Q_10_, the temperature sensitivity coefficient of ecosystem respiration.

**Figure 9:**
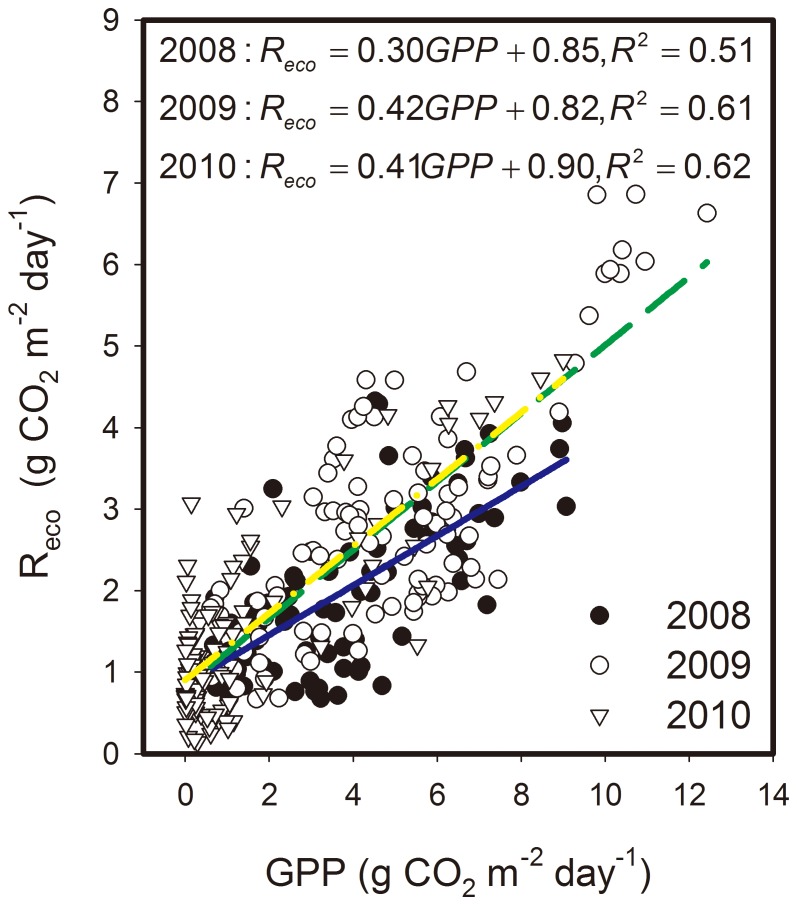
Ecosystem respiration (R_eco_) responses to gross primary production (GPP) in a linear manner.

The daily R_eco_ values were positively linearly correlated with the daily GPP values, and more than 50% of the variations in R_eco_ can be explained by GPP in the three growing seasons (*P*<0.01, Figure 9). The coefficient of determination (R^2^) between R_eco_ and GPP in 2008 (dry soil water conditions) was obviously lower than that in 2009 and 2010 (wet soil water conditions).

### 2.4. Response of Carbon Fluxes to LAI

**Figure 10:**
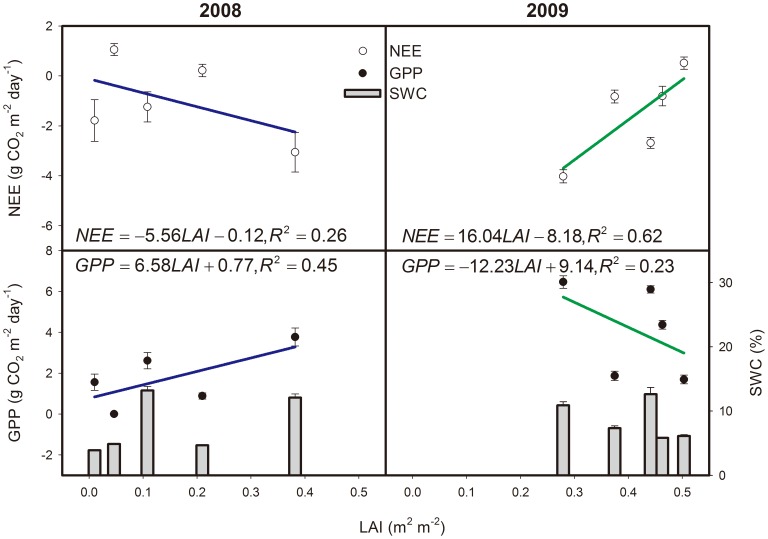
Relationships between daily net ecosystem CO_2_ exchange (NEE), daily gross primary production (GPP), and leaf area index (LAI). NEE, GPP, and soil water content (SWC) data represent the seven-day mean that is centered on the day samples for LAI. Error bars represent one standard error.

The LAI in the growing seasons of 2008 and 2009 were measured. In 2008, changes in LAI explained 45% of the variations in GPP, with the increase in GPP per unit LAI of 6.58 g CO_2_ m^−2^ day^−1^. On the other hand, the linear relationship between GPP and LAI in 2009 changed into a negative relation (Figure 10). The relationship between NEE and LAI yielded similar results. Relatively high LAI was observed in July and August 2009 despite a severe drought in the temperate desert steppe (Figure 10).

## Discussion

### 3.1. Magnitude of Carbon Flux Compared with Other Grassland Ecosystems

The temperate desert steppe ecosystem accumulated a total of 4.2 g C m^−2^ from 2008 to 2010, averaging 1.4 (±25.0, S.D.) g C m^−2^ yr^−1^ annually, suggesting that it is neutral. Large interannual variability in NEE can also be found in literature on grassland ecosystems in the Inner Mongolia and the Mongolian Plateau (Table S1). For example, net carbon loss was reported in the Inner Mongolia *Stipa Krylovii* steppe [49] and the Inner Mongolia *Leymus Chinensis* steppe [27], while a significant carbon sink was reported for the grazed typical steppe in Central Mongolia [50]. In terms of other grassland ecosystem types, some studies reported a carbon sink in the alpine meadow-steppe in the Qinghai-Tibet Plateau [51], [52], while Fu *et al.* [27] found the opposite. For European grasslands, Hussain *et al.* [22] recently reported that the managed grassland in Germany was a carbon sink. Based on the EUROGRASSFLUX data, Gilmanov *et al.* [53] found that four out of nineteen cases of grasslands did not perform as annual net CO_2_ sinks. For Mediterranean climate grasslands [5], [11], [17], the ecosystem could be a carbon source or a carbon sink depending on the precipitation quantity and the timing of rain events. Such alternations between carbon sink and carbon source have been reported on a Canadian temperate grassland [12], a semi-desert grassland in the USA [23]. Due to the seasonal drought and overgrazing, a tropical pasture in Panama was a strong carbon source [54]. Some North American prairies had characteristics similar to a consistent carbon sink [55]. However, fire burning in the spring could result in net carbon loss in the Oklahoma native tallgrass prairie [16]. In general, as shown through the literature cited above, the annual NEE of grassland ecosystems can be a carbon sink or carbon source among different hydrological years. The lower than normal precipitation or drought and management could be the primary factors that lead to the carbon source for grassland ecosystems.

### 3.2. Environmental Regulation of Carbon Fluxes

#### 3.2.1. Effects of drought on GPP and R_eco_

Reichstein *et al.* [18] hypothesized that short-term drought would suppress R_eco_ more than GPP because litter and upper soil layers that dry first are the locations of most heterotrophic respiration, whereas photosynthesis could be supported by moisture that is accessible to the roots in deeper soil layers. In the temperate desert steppe studied here, the drought suppressed GPP more than R_eco_ during the three study years. The root distribution of these grasslands plants was mainly in the soil layer less than 0.30 m depth [56]. Yang *et al.* [57] reported that SWC deeper than 0.40 m always remained at a near-constant value, and deeper ground water discharged for assimilation was very limited in the temperate desert steppe. Consequently, the impact of the drought might have lasted longer for assimilation than respiration in this ecosystem.

#### 3.2.2. Effects of environmental variables on R_eco_

Q_10_ in 2010 was lower than that in 2008, although the soil water content in 2010 was higher than that in 2008. The SWC sensitivity of R_eco_ for different hydrological years in the temperate desert steppe was also found to be not consistent with the hypotheses that SWC would constrain R_eco_ more during a dry year than during a wet year [18]. The discrepancy indicated that beyond soil temperature and moisture which is widely accepted [58], some others may make also important influence on R_eco_, such as biotic factors (photosynthesis or substrate availability) [59]–[61]. The data of the current study suggest that GPP may have mediated R_eco_, which responds more to GPP under wet soil conditions than under dry soil conditions. Based on the results derived from the water addition field experiment, Yan *et al.* [29] also demonstrated that photosynthetic substrate supply was an important factor in regulating soil respiration in both daily and seasonal timescales in semiarid steppe ecosystems and that its effect on R_eco_ increased with increasing water availability. A strong linear relationship between R_eco_ and GPP was also reported in the Mediterranean C3/C4 grassland [11], [17] and oak–grass savannah [62]. However, recent results have shown that respiration might be partly driven by GPP, and the respiration components, including autotrophic and heterotrophic maybe partly dependent on the respective substrate availability [8]. Bahn *et al.* [26] and Yan *et al.* [29] reported that the response of R_eco_ to the changes in photosynthesis has a significant time lag of zero to three hours in the grassland ecosystem. Although it may be challenging, the modulation of photosynthesis on respiration should be incorporated as a physiological process to mechanism-based carbon model [63].

#### 3.2.3. Effects of LAI and SWC on carbon Flux

The linear relationship between carbon fluxes and LAI has been demonstrated in the grassland in central Japan [64], Mediterranean climate grassland [5], [11], [17], Canadian temperate grassland [12], and Inner Mongolia *Leymus chinensis* steppe [27]. The low LAI explanation for the variance of NEE (26%) or GPP (53%) for the Mongolia typical steppe [50], and the poor relationship between carbon flux and LAI for the Inner Mongolia typical *Stipa krylovii* steppe have also been reported [49]. Obvious discrepancy in the relationship between carbon flux and LAI in the temperate desert steppe was observed for the two precipitation pattern years. The SWC variance might be the primary factor that affects the response of carbon flux to LAI. A good relationship between carbon flux and LAI can be achieved under sufficient water conditions. However, the vegetation function structural processes, such as LAI, respond to drought stress later than the physiological or biochemical processes, such as vegetation photosynthesis and microbial respiration, which were strongly related to the ecosystem carbon flux. The relationship between carbon flux and LAI may be weak under drought stress conditions, even the possibility of high LAI value. Thus, we suggest that SWC might have the same importance as LAI on the ecosystem carbon uptake in the temperate desert steppe characterized by drought, and it might become dominant under specific conditions.

#### 3.2.4. Effects of seasonal precipitation pattern on carbon balance

Rainfall seasonal variability alters ecosystem carbon dynamics, regardless of the amount of total annual precipitation [8], [65]. In terms of temperate desert steppe, the annual precipitation in 2010 was similar to that in 2008, whereas the precipitation in July and August was significantly lower than that in 2008, leading to a drop in SWC, a decline in photosynthetic activity, and a lower absolute cumulative NEE in 2010. High precipitation in September 2010 could not offset the reductions caused by the severe summer drought. Jongen *et al.* [5] also found that the lack of precipitation at the peak growth could result in a decrease in annual carbon sequestration in the Mediterranean grassland.

Precipitation variance has also been found to influence the timing and duration of canopy development and, therefore, regulate the biomass production on northern mixed-grass prairies [66]–[68]. Functional changes in grass vegetation could affect the biological and ecological processes, such as stomatal conductance, photosynthesis, and respiration. The long summer drought period in 2010 could have induced the functional change in vegetation in the temperate desert steppe, resulting in the shorter length of the carbon sink days of 2010. The shallow roots of grass species were mostly distributed lower than 50 cm, and soil water availability was dependent on the atmospheric rainfall rather than on the deep groundwater supply [69]. Plant community structure may be sensitive to rainfall variability, especially to seasonal drought stress, which may have an indirect effect on carbon flux [8].

Xu *et al.* [17] reported that the timing of rain events had more impact than the total amount of precipitation on the ecosystem NEE for the Mediterranean grassland. Similar results were also found for the savannah ecosystem [62] and alpine shrub lands in Qinghai-Tibetan plateau [52]. However, the NEE in the temperate desert steppe was much larger in 2009, with more annual precipitation, than in 2008 and 2010, which had low annual precipitation. These results suggest that carbon sequestration in the temperate desert steppe primarily depends on the amount of annual precipitation and the summer precipitation quantity. Hence, the summer drought could have a significant influence on the annual carbon balance.

### Conclusions

The temperate desert steppe ecosystem was close to carbon neutrality during 2008 to 2010. The seasonal variation in NEE was correlated with rain events and soil moisture. This study demonstrated that the carbon sequestration capacity of the temperate desert steppe is sensitive to changes in the precipitation seasonal distribution rather than just the annual precipitation quantity. Summer drought stress could influence the annual carbon balance.

Furthermore, the drought suppressed GPP more than R_eco_ and the SWC sensitivity of GPP was greater during the drought year. The photosynthetic substrate supply displayed an important role in regulating respiration on the daily timescale but the magnitude of this effect became less apparent during the drought year, when there was limited soil moisture availability.

## Supporting Information

Table S1 **Comparison of annual net ecosystem CO_2_ exchange (NEE) among different temperate grassland ecosystems.**
(DOC)
