# Supplementary material for: Sensitivity of Temperate Desert Steppe Carbon Exchange to Seasonal Droughts and Precipitation Variations in Inner Mongolia, China
Source: PLoS One. 2013 Feb 5;8(2):e55418. doi: 10.1371/journal.pone.0055418 (PMC3564909; doi:10.1371/journal.pone.0055418)
Supplement: Table S1 — Comparison of annual net ecosystem CO2 exchange (NEE) among different temperate grassland ecosystems. (DOC) [file pone.0055418.s001.doc]

**Table S1** Comparison of annual net ecosystem CO_2_ exchange (NEE) among different temperate grassland ecosystems.

| Grassland type | Location | Climate | Management | PPT_m_^1^ | Study period | PPT^2^ | Annual NEE^3^ |
| --- | --- | --- | --- | --- | --- | --- | --- |
| Temperate dessert steppe^4^ | China  (44°05'N; 113°34'E) | Temperate continental climate | Fenced | 184 | 2008  2009  2010 | 136  186  141 | -7.2  -22.9  26.0 |
| Typical steppe [49] | China  (44°08'N; 116°20'E) | Temperate continental climate | Fenced | 290 | 2004  2005  2006 | 297  174  215 | 51.0^5^ |
| Typical steppe [27] | China  (44°32'N; 116°40'E) | Temperate continental climate | Fenced | 350 | 2004  2005 | 364  153 | 106.9  139.9 |
| Steppe [50] | Mongolia  (47°13'N, 108°44'E) | Temperate continental climate | Grazed | 196 | 25 March 2003 to  24 March 2004 | 248 | -41.0 |
| Temperate grassland [12] | Canada  (49°26'N, 112°34'W) | / | Not grazed | 402 | 1999  2000 | 314  276 | -20.6  18.1 |
| Semidesert grassland [23] | USA  (31°44'N, 109°56'W) | / | Grazed | 345 | 2005  2006  2007  2008  2009 | 162  274  313  312  246 | 21.0  -55.0  -69.0  -98.0  -47.0 |
| Native tallgrass prairie [55] | USA  (36°56'N, 96°41'W) | / | Not grazed;  Burned in the spring | 976 | 31 March 1997 to  30 March 1998 | 1134 | -268.0 |
| Native tallgrass prairie [16] | USA  (36°56'N, 96°41'W) | / | Not grazed;  Burned in the spring | / | 1997  1998  1999 | / | -4.0  151.0  30.0 |
| Temperate grassland [22] | Germany  (50°58'N; 13°34'E) | Humid continental climate | Extensively managed | 824 | 2003  2004 | 512  798 | -295.0  -260.0 |
| European grasslands [53] | 20 various sites | Wide range of climatic conditions | Grazed; Cut | 450~1816 | Various years | / | -171.0~652.9 |

^1^PPT_m_, mean annual precipitation (mm);

^2^PPT, annual precipitation during study year (mm);

^3^Annual NEE^3^, annual net ecosystem CO_2_ exchange during study year (g C m^-2^ yr^−1^);

^4^This study;

^5^The value was the average of three year.
